# Preparation of NLCs-Based Topical Erythromycin Gel: In Vitro Characterization and Antibacterial Assessment

**DOI:** 10.3390/gels8020116

**Published:** 2022-02-13

**Authors:** Ameeduzzafar Zafar, Syed Sarim Imam, Mohd Yasir, Nabil K. Alruwaili, Omar Awad Alsaidan, Musarrat Husain Warsi, Shehla Nasar Mir Najib Ullah, Sultan Alshehri, Mohammed M. Ghoneim

**Affiliations:** 1 Department of Pharmaceutics, College of Pharmacy, Jouf University, Sakaka 72341, Al-Jouf, Saudi Arabia; nkalruwaili@ju.edu.sa (N.K.A.); osaidan@ju.edu.sa (O.A.A.); 2 Department of Pharmaceutics, College of Pharmacy, King Saud University, Riyadh 11451, Riyadh, Saudi Arabia; salshehri1@ksu.edu.sa; 3 Department of Pharmacy, College of Health Science, Arsi University, Asella 396, Ethiopia; mohdyasir31@gmail.com; 4 Department of Pharmaceutics and Industrial Pharmacy, College of Pharmacy, Taif University, Taif 21944, Makkah, Saudi Arabia; mvarsi@tu.edu.sa; 5 Department of Pharmacognosy, College of Pharmacy, King Khalid University, Abha 61421, ’Asir, Saudi Arabia; shehlanasar2005@gmail.com; 6 Department of Pharmacy Practice, College of Pharmacy, Almaarefa University, Ad Diriyah 13713, Ar Riyad, Saudi Arabia; mghoneim@mcst.edu.sa

**Keywords:** erythromycin, ocular delivery, nanostructured lipid carrier, corneal permeation, antibacterial study

## Abstract

In the present study, erythromycin (EM)-loaded nanostructured lipid carriers (NLCs) were prepared by the emulsification and ultra-sonication method. EM-NLCs were optimized by central composite design using the lipid (A), pluronic F127 (B) and sonication time (C) as independent variables. Their effects were evaluated on particle size (Y_1_) and entrapment efficiency (Y_2_). The optimized formulation (EM-NLCs-opt) showed a particle size of 169.6 ± 4.8 nm and entrapment efficiency of 81.7 ± 1.4%. EM-NLCs-opt further transformed into an in-situ gel system by using the carbopol 940 and chitosan blend as a gelling agent. The optimized EM-NLCs in situ gel (EM-NLCs-opt-IG4) showed quick gelation and were found to be stable for more than 24 h. EM-NLCs-opt-IG4 showed prolonged drug release compared to EM in situ gel. It also revealed significant high permeation (56.72%) and flux (1.51-fold) than EM in situ gel. The irritation and hydration study results depicted no damage to the goat cornea. HET-CAM results also confirmed its non-irritant potential (zero score). EM-NLCs-opt-IG4 was found to be isotonic and also showed significantly (*p* < 0.05) higher antimicrobial activity than EM in situ gel. The findings of the study concluded that NLCs laden in situ gel is an alternative delivery of erythromycin for the treatment of bacterial conjunctivitis.

## 1. Introduction

The eye is the most sensitive organ of the body and problems associated with the eye directly affect vision. Most ocular illnesses, such as bacterial conjunctivitis, sympathetic ophthalmia, uveitis and retinal disease, are treated by the conventional ophthalmic eye drop. However, the eye drop has drawbacks, such as less ocular residence time and quick elimination from the ocular region. Only 5% of the administered dose is available for pharmacological activity due to the elimination of the administered dose in 2 min from the corneal surface by blinking, dilution with tear fluid, as well nasolacrimal drainage duct [1,2]. Therefore, efforts have been taken to enhance the ocular residence time, in order to achieve drug permeability. Viscous preparation, such as with conventional gel and ointment, enhances residence time on the eye surface, but has drawbacks such as stickiness, blurred vision, reflex blinking, as well as irritation [3,4]. The novel approach to developing a formulation with reduced administration frequency and greater residence time leads to an increase in patient compliance. Different novel formulations, including nanostructured lipid carriers (NLCs) [5], liposomes [6], niosomes [7], ocular inserts containing nanoparticles [8] and in situ gel systems [9], have been reported to increase bioavailability as well as therapeutic efficacy. These formulations have been reported to increase drug accumulation and retention at the target site [10].

Among these formulations, NLCs are widely reported ophthalmic formulations, due to their stability in the physiological environment. They consist of biocompatible solid and liquid lipids, as well as surfactants. They are also reported for high drug load without drug leakage [5], which enhances drug release and bioavailability [11]. Various studies published on ocular NLCs report a high corneal contact time in comparison to pure drug solution [5,12,13,14]. They report a significant enhancement in corneal permeation and a decrease in ocular clearance. The incorporation of NLCs into an in-situ gel system (stimuli sensitive agent) can further improve drug release, contact time, and minimize toxicity and stability. Research regarding the ciprofloxacin moxifloxacin and sertaconazole in situ gel system report prolonged release, residence time and increases in bioavailability and antimicrobial activity [15,16,17].

Erythromycin (EM) is a macrolide category antibiotic having broad-spectrum antimicrobial activity. It penetrates the bacterial cell membrane, binds to the ribosome (50s) reversibly, and stops protein synthesis. EM is also administered new-born babies to prevent ocular infection [18,19].

CP is polyacrylic acid in nature, biocompatible, non-toxic and a good bioadhesive polymer able to easily transform to gel from the sol system in a pH environment [20]. It also binds to the mucin by hydrogen bond and electrostatic force and improves ocular residence time [21]. Despite possessing outstanding, mucoadhesive properties, it may produce irritation and may damage the ocular tissue [21]. Therefore, a combination of CP with CS overcomes this limitation [22]. CS is a natural, biocompatible, biodegradable, bioadhesive polymer, and also exhibits antimicrobial activity [23,24]. There is no early research reporting on the EM-NLCs in situ gel system using the polymer blend CP and CS.

The present research is designed to develop EM-NLCs in situ gel using polymers such as carbopol (CP) and chitosan (CS) in different ratios. EM-NLCs were optimized by central composite experiment design using the solid lipid, liquid lipid and surfactant. The optimized formulation (EM-NLCs-opt) was further loaded into an in-situ gel (IG) system and then evaluated for gelling capacity, clarity, viscosity and pH. Finally, it was evaluated for in vitro drug release, mucoadhesive study, trans-corneal permeation, hydration study, ocular irritation, isotonicity and antimicrobial study.

## 2. Materials

Erythromycin (EM) was procured from Envee Drugs Pvt Ltd. (Gandhinagar, India) from India. Stearic acid, Compritol 888, Isopropyl myristate, Myristic acid, Lauric acid and Cetyl palmitate Pluronic F127, tween 20, Cremophor EL, Bile Salt, Span 20, chitosan and acetonitrile dialysis bag (MWCO 12kDa) were procured from Sigma Aldrich (St. Louis, MO, USA). Oleic acid, Peceol, sesame oil, Soya oil, olive oil, and coconut oil were procured from Loba Chemie (Mumbai, India). Carbopol 940 was obtained from Acros Organic (Newark, NJ, USA). Other chemicals used for study are analytical grade.

## 3. Experimental

### 3.1. Screening of Excipients

Selection of solid lipid, liquid lipid and surfactant was carried out based on the maximum solubility of EM. The excess amount of EM was dissolved in melted solid lipids (2 mL of stearic acid, compritol 888, isopropyl myristate, myristic acid, lauric acid and cetyl palmitate). The mixture was vortexed (2 min) and kept in a water bath shaker for 72 h to achieve supersaturation. The different liquid lipids—oleic acid, peceol, sesame oil, soya oil, olive oil, and coconut oil (1 mL) were taken in glass vials. The excess amount of EM was added to each oil and vortexed for 5 min. Then, it was kept in an orbital shaker for 72 h at room temperature. Selection of surfactants was carried by adding the excess amount of EM to different surfactants, pluronic F127, tween 20, cremophor EL, bile salt, and span 20 (1 mL), vortexed and then kept in an orbital shaker for 72 h. All the tested samples were centrifuged at 6000 rpm for 30 min to separate the insoluble EM. The supernatant was collected and analysed for drug concentration by UV-Visible spectrophotometer (Genesys 10S UV-Vis, Thermo-scientific, Waltham, MA, USA) at 280 nm.

### 3.2. Selection Solid and Liquid Lipid Ratio by Miscibility

The selected solid and liquid lipids were mixed in different ratios (9:1, 8:2, 7:3, 6:4, 5:5) and miscibility was evaluated visually. The mixture was heated at 60 °C for 25 min and kept aside for cooling to room temperature. The mixture was assessed visually for phase separation and turbidity [25]. 

### 3.3. Optimization

The optimization of EM-NLCs was performed by using central composite design (CCD; Design-Expert^®^ software, version 8.0.6, Stat-Ease, Minneapolis, MN, USA) to evaluate the influence of independent parameters (lipid, surfactant, sonication time) on dependent parameters particle size (nm, PS) and entrapment efficiency (%, EE). CCD is a type of factorial or fractional factorial design with centre points, amplified with a group of axial points (also called star points). CCD works on the principal of 8 factorial points, 6-star points, and 6 replicated centre points (for the statistical assessment), which were used for optimization [26]. As shown in Table 1, lipid (%, A), surfactant (%, B) and sonication time (min, C) were chosen as independent factors and their effect was observed on PS (nm, Y_1_) and EE (%, Y_2_). The design depicted the total number of runs of 2k + 2k + n, where k is the number of independent variables and n is the number of repetitions of experiments at the centre point [27]. For the prediction of the best formulation, the fitness of the model due to the analysis of variance was detected among the linear, two-factor (2F) interaction model and quadratic model [28]. For optimization, the focus was made to maximize the correlation coefficient value (R^2^), i.e., multiple correlation coefficient, predicted correlation coefficient and adjusted correlation coefficient. The optimization was made by the point prediction method using the desirability function to obtain the minimum PS and maximum EE. The effect of selected independent factors on the dependent factors was assessed by the 3D images and polynomial equations obtained from the software [29]. The mathematical format of the polynomial equation was represented by equation (1):

Y = β_0_ + β_1_ A + β_2_ B + β_3_ C + β_12_ AB + β_13_ AC + β_23_ BC + β_11_ A^2^ + β_22_ B^2^ + β_33_ C^2^ (1)

Y, representing the theoretical value, β_0_ is the intercept, β_1_, β_2,_ β_3,_ represents the model coefficient. The statistical tests such as ANOVA and lack of fit test were carried out to determine the statistically significant model. 

### 3.4. Formulation of Erythromycin Nanostructured Lipid Carrier (EM-NLCs)

EM-NLCs were prepared by the previously reported emulsification ultra-sonication method with slight modification [30]. The detailed composition of the formulations (EM-NLCs1—EM-NLCs20) is shown in Table 2. The solid lipid was melted at 80 °C (5 °C above the melting point). The liquid lipid and EM were added into melted solid lipid to form a homogenous mixture. Separately, a hot aqueous surfactant solution was prepared and heated to the same temperature (85 °C). The surfactant solution was added to the molten lipid mixture with continuous stirring to form the pre-emulsion. The prepared pre-emulsion homogenized (IKA T50 digital Homogenizer, Germany) at 7000 rpm for 2 min and then subjected to ultrasound using a probe sonicator (Qsonica-500 Sonicator, Newtown, CT, USA). The formed nanoemulsion was transferred to glass vials and kept at room temperature (25 °C) for recrystallization to form NLCs. The prepared EM-NLCs were stored in the vial for further characterization. 

### 3.5. Characterization 

#### 3.5.1. NLCs Evaluation 

The particle size (PS), polydispersibility index (PDI), and zeta potential (ZP) were measured by the size analyzer (Malvern zeta sizer, Malvern, UK). The diluted samples (100-fold) were placed into the cuvette and analysed for PS and PDI. ZP was measured by taking the samples into a cuvette having an electrode. Each sample was analysed in triplicate and data were shown as mean ± SD. 

#### 3.5.2. Entrapment Efficiency (EE)

The entrapment efficiency of EM-NLCs was analysed by the indirect method. The prepared EM-NLCs were filled into a centrifugation tube and centrifuged at 10,000 rpm into a centrifuge (Hettich, Tuttlingen, Germany). The supernatant was separated, and the EM content was analysed by UV spectrophotometer (Genesys 10S UV-Vis, Thermo-scientific, Waltham, MA, USA) at 280 nm. Then, the amount of EM entrapped into NLCs formulation was calculated by the below formula:

(2) $$\mathrm{EE}(\%)=\frac{\mathrm{Total}\;\mathrm{EM}-\mathrm{Unentrapped}\;\mathrm{EM}}{\mathrm{Total}\;\mathrm{EM}}\times 100$$

#### 3.5.3. Development of In Situ Gel of EM-NLCs (EM-NLCs-IG)

CCD optimized composition (EM-NLCs-IG) was converted to in situ gel (EM-NLCs-IG) by using the polymer blend chitosan and Carbopol 940. The fixed concentration of chitosan (0.4% *w*/*v*) was transferred to acetate buffer (pH 4.5) and kept overnight with stirring. The various concentrations of carbopol solution were prepared by dispersing in distilled water, as shown in Table 3. The chitosan solution (0.4% *w*/*v*) was added to carbopol solution and mixed to obtain a homogeneous dispersion. Finally, the lyophilized EM-NLCs (0.5%) were dispersed into carbopol-chitosan solution and stored for further study.

#### 3.5.4. Gelling Strength and Viscosity

The gelling strength of EM-NLCs-IG was evaluated using simulated tear fluid (STF, sodium chloride, sodium bicarbonate, and dehydrated calcium chloride). STF (2 mL) was taken into a glass vial and a drop of EM-NLCs-IG (sol form) was added. The temperature was maintained at 37 ± 0.5 °C and visually evaluated for gelling time, as well as to convert again into sol form [31]. The viscosity of EM-NLCs-IG was analysed by the Brookfield viscometer (Fungilab, Barcelona, Spain) at 20 rpm.

#### 3.5.5. Clarity, Optical Transmittance and pH Determination

The clarity of ophthalmic preparation was necessary due to the presence of visible particles that irritate the ocular tissue. The clarity was examined visually by keeping the sample behind the white backboard in sol as well as gel state. The transmitting of EM-NLCs-IG was examined by UV spectrophotometer at 480 nm using STF as blank. The pH of EM-NLCs-IGs was measured by pH meter (Digital pH meter, Hicon, New Delhi, India). 

#### 3.5.6. In Vitro Drug Release

The in vitro release of EM from EM-NLCs-opt, EM-NLCs-opt-IG4 and EM in situ gel (EM-IG) having the same dose of EM were analysed by the pre-treated dialysis bag method. The samples (1 mL; 0.5%) were transferred to a dialysis bag (molecular weight cut off, 12–14,000 kDa, Sigma Aldrich, St. Louis, MO, USA) and immersed into STF (100 mL), as release media. The release assembly was fixed on the thermostat magnetic stirrer with 100 rpm and a temperature of 37 ± 0.5 °C. At a predetermined interval, 2 mL of release media was taken and replaced with fresh STF to maintain the concentration gradient. The released content was assessed on the UV-Vis spectrophotometer at 280 nm after appropriated dilution. The release value of the optimized EM-NLCs-opt-IG4 was fitted into various kinetic release models for finding the release pattern of EM. 

#### 3.5.7. Mucoadhesive Study 

The mucoadhesive potential of EM-NLCs-opt-IG4 was performed by the physical balance method using goat cornea [32,33]. The freshly excised goat cornea was collected from the slaughterhouse and washed to remove adhered materials. Then, it was attached to the backside of the physical balance pan and fixed to the sample holder. The temperature was maintained at 37 ± 0.5 °C for the whole study. The sample was transferred to the sample holder and kept for a few minutes to attach to the cornea. The weight was added to the second pan of physical balance until the cornea was separated from the EM-NLCs-opt-IG4. The weight at which the sample separated from the balance is noted and bioadhesive potential was calculated by the given formula and denoted by dyne/cm^2^.

#### 3.5.8. Ex Vivo Goat Corneal Permeation

The freshly excised goat cornea was used for the permeation study and the result was compared to the amount of EM permeated from EM-NLCs-opt, EM-NLCs-opt-IG and EM-IG. The fresh eyeball was collected from a local slaughterhouse and stored in 0.9% NaCl solution at 4 °C. The cornea was separated with the sclera and washed with STF. The cornea was mounted between the donor and acceptor compartment of the diffusion cell (Franz diffusion, Logan FDC-6, Hudson, NJ, USA). The receptor media (STF) was filled to the acceptor compartment and the temperature was maintained at 37 ± 0.5 °C. The samples (equivalent to 0.5% EM) were filled into the donor compartment and at a definite time, the permeated sample (1 mL) was collected. The same volume of fresh blank STF was added to maintain the uniform volume. The sample was filleted through a filter (0.45 µm), diluted and then injected (20 µL) into the HPLC column for the analysis using the previously developed HPLC method [34]. HPLC instrument (HPLC, Auto sampler, Shimadzu LC10AD, Kyoto, Japan) with C18 column, mobile phase system 0.02 M potassium phosphate buffer and acetonitrile (60:40), a flow rate of 0.75 mL/min and detection at 254 nm was employed for the evaluation. The amount of drug permeated and flux was calculated. 

#### 3.5.9. Histopathological Examination

This study was carried out to observe any alteration in the internal corneal structure with the tested samples compared with the positive control. The tissue was treated similar to the ex vivo corneal permeation study for EM-NLCs-opt-IG4, negative control (0.9% NaCl) and positive control (1% *w*/*v*; SLS). The cornea was collected and stored in formalin solution (0.8%, *v*/*v*). The cornea was fixed into the solid block using paraffin after dehydration with alcohol. The microtome cutter was used to cut the cross-section and further stained with haematoxylin and eosin dye. The image was captured with a Motic digital optical microscope at 40× magnification. 

#### 3.5.10. Corneal Hydration

The corneal hydration test was performed to determine the ocular tolerance of the cornea [35]. The goat cornea was taken after the permeation study and the initial weight was taken (W_1_, wet weight). Then, it was placed into a hot air oven at 60 °C for 72 h and again weighted (W_2_, dry weight). The corneal hydration (%) was calculated as per the given equation (Equation (3))

(3) $$\mathrm{Corneal}\;\mathrm{hydration}\;(\%)=\frac{W1-W2}{W2}\times 100$$

#### 3.5.11. HET-CAM Irritation Study

HET-CAM study is an in vitro method used to determine the irritation potential of the selected formulation (EM-NLCs-opt-IG4) and the result was compared with normal saline (negative control) and 0.1 N NaOH (positive control). The study was performed on chorioallantoic membrane (CAM) of a hen’s egg, which is similar to the blood capillary of the eye [36]. The fertilized eggs were collected from a local poultry farm and placed in a box with the air chamber in an upward position. The eggs were incubated for nine days at 37 ± 1 °C and 50 ± 1% RH (Orbital incubator, Thermo Fisher Scientific, Waltham, MA, USA). On the 10th day, the eggs were removed from the incubator and shell was removed using forceps. The normal saline was added to remove white membrane without damaging the CAM. The samples of EM-NLCs-opt-IG4, negative control and positive control were added and the irritation scores were noted at a different time from the scale (0–0.9 non-irritant; 1–1.99 mild irritant; 2–2.99 moderate irritant; 3–<3 severe irritant) [37].

#### 3.5.12. Sterility and Isotonicity Evaluation 

Sterility was evaluated by using fluid thioglycollate and soybean casein digest medium. The fluid thioglycollate and soybean casein digest medium were prepared and sterilized at 121 °C for 15 min in an autoclave. The sample (EM-NLCs-opt-IG4) was inoculated in both mediums under aseptic conditions then incubated for 14 days and evaluated under aseptic conditions for turbidity and precipitation. Isotonicity of the EM-NLCs-opt-IG4 was evaluated on the goat blood sample and the result was compared with normal saline (0.9% NaCl). A drop of blood was taken on a glass slide and mixed with EM-NLCs-opt-IG4 and normal saline. The smear was prepared on a glass slide and further stained with Leishman’s stain. The slide was kept aside to air-dry and the damage in red blood cells was observed under the microscope [38].

#### 3.5.13. Antibacterial Activity 

The antimicrobial susceptibility test of the developed formulation (EM-NLCs-opt, EM-NLCs-opt-IG4, EM-IG4) was evaluated by disk diffusion method on Gram-positive (*Staphylococcus aureus*) and Gram-negative (*Escherichia coli*) in nutrient agar medium. The bacterial strain was grown on the sterile Luria Bertani broth. The nutrient agar medium was prepared and sterilized at 121 °C. The bacterial culture of both microorganisms (100 µL, 10^6^ CFU/mL) was mixed with the agar medium (10 mL), transferred into a Petri dish under aseptic conditions, and kept for solidification without any agitation. The samples (EM-NLCs-opt-IG4 and EM-IG) were soaked into a disk and placed in a Petri plate under aseptic conditions. The plate was placed into the incubator for 24 h in an inverted position and the zone of inhibition was measured for each sample by the graduated scale and the result was compared.

## 4. Result and Discussion

### 4.1. Screening of Solid and Liquid Lipid

**Figure 1:**
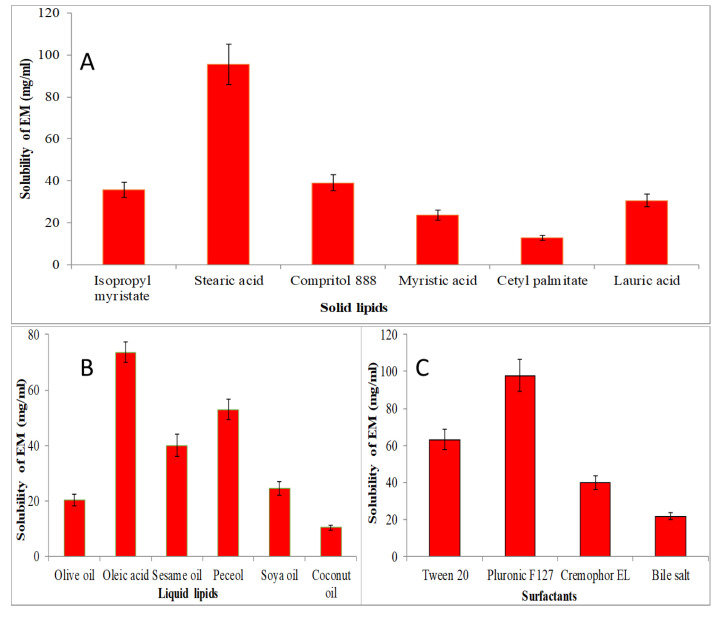
Solubility of EM in various solid lipids (**A**), liquid lipids (**B**), and surfactant (**C**). The study performed in triplicate and data shown as mean ± SD.

The screening of solid lipid, liquid lipid and surfactant were performed for the selection of lipid and surfactant based on the maximum solubility, and results are expressed graphically in Figure 1. The solubility order of EM in solid lipids was found to be as stearic acid > compritol 888 > isopropyl myristate > myristic acid > lauric acid > cetyl palmitate (Figure 1A). The maximum solubility of EM was found to be in stearic acid (95.5 ± 9.5 mg/mL). EM showed solubility order in different liquid lipids as oleic acid > peceol > sesame oil > soya oil > olive oil > coconut oil (Figure 1B). The maximum solubility of EM was found to be in oleic acid (73.63 ± 3.65 mg/mL). Figure 1C shows the solubility of EM in various surfactants in order as pluronic F127 > tween 20 > cremophor EL > bile salt > span 20. The maximum solubility of EM was found in pluronic F127 as 97.87 ± 8.81 mg/mL. So, finally, stearic acid, oleic acid and pluronic F127 were selected as the final ingredients for the formulation of NLCs.

### 4.2. Selection Solid and Liquid Lipid Ratio by Miscibility

The solid (stearic acid) and liquid lipid (oleic acid) were mixed in various ratios (9:1, 8:2, 7:3, 6:4, 5:5) and visually observed for phase separation and turbidity. The final ratio of solid and liquid lipid at 6:4 exhibited no phase separation, turbidity as well as did not show any separated oil droplets on the filter paper. 

### 4.3. Optimization

The formulation design is an important factor to consider the effect of factors, in addition to the interactions. In the present study, lipid (%, A), surfactant (%, B) and sonication time (min, C) were chosen as independent factors and their effect was observed on PS (nm, Y_1_) and EE (%, Y_2_) (Table 2).

### 4.4. Effect of Variables on Size (Y_1_)

**Figure 2:**
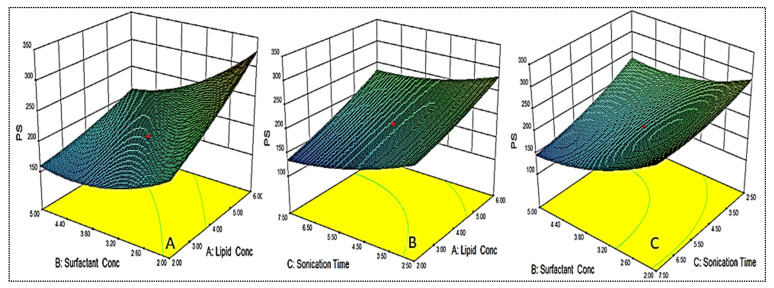
3D response surface plot showing effect of factors lipid (**A**), surfactant (**B**) and sonication time (**C**) on the particle size (Y_1_).

As shown in Table 2, the PS for different formulations were found in the range of 122.6 ± 6.1 nm (EM-NLCs7) to 350.6 ± 4.2 nm (EM-NLCs2). The lowest PS was found using lipid (6%), surfactant (2%) and sonication time (2.5 min). The highest PS shown by the formulation with lipid composition (2%), surfactant (5%) and sonication time (7.5 min). The result exhibited a significant variation in PS after varying the composition. The used three factors showed a considerable effect on the PS. The three-dimensional response surface plot (Figure 2A) and polynomial Equation (4) depicted the effect of independent variables on the PS (Y_1_). The factors, i.e., lipid (A) showed a positive effect, surfactant (B) and sonication time (C) showed a negative effect on the PS. The increase in PS was observed with the increase in lipid content, as shown in EM-NLCs1 to EM-NLCs2 (Table 2). This might be due to the presence of excess lipids, which increase the viscosity of the dispersion [27]. The other reason may be incomplete emulsification, which may be responsible for the aggregation of the particle due to the lack of surfactant. The surfactant (B) demonstrated a negative effect on PS due to the reduction in interfacial tension between the aqueous and lipid phase, resulting in the reduction of PS (EM-NLCs1-EM-NLCs3). A similar type of finding was reported by Yang et al. in their research [39]. The third factor sonication time (C) also showed a negative effect. On increasing the duration of sonication, the PS of NLCs was decreased (EM-NLCs1- EM-NLCs5). This might be due to the increase in the energy to the system, which is responsible for the prevention of particle agglomeration [40]. The combined effect of the factors also depicted a significant effect on the PS. The second-order polynomial generated for PS is given below:

Particle size (Y_1_) = 195.43 + 42.85A − 40.95B − 30.95C − 17.55AB + 6.88AC − 14.55BC + 6.63A^2^ + 24.01B^2^ + 13.53C^2^ (4)

The data were fitted to various design models and the best fit model was found to be quadratic. The regression value (R^2^ = 0.9827) was found to be maximum for this model. The lack of fit was found to be insignificant (*p* = 0.6787). The regression values of the selected responses during optimization were given in Table 3. The regression coefficient (R^2^) of this equation was found to be 0.9827, indicating the good correlation between response and selected factors.

### 4.5. Effect of Variables on Encapsulation Efficiency

**Figure 3:**
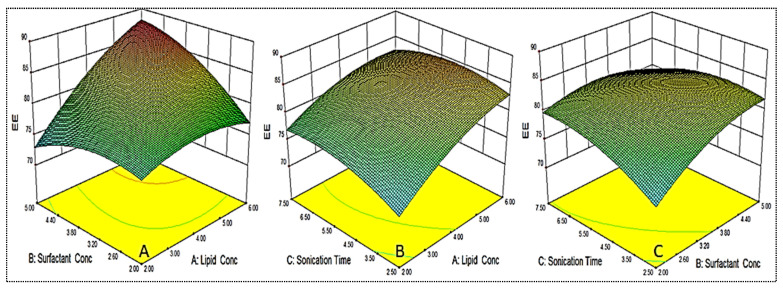
3D response surface plot showing effect of factors lipid (**A**), surfactant (**B**) and sonication time (**C**) on the encapsulation efficiency (Y_2_).

As shown in Table 2, the EE for different formulations was found in the range of 69.4 ± 1.3% (EM-NLCs2) to 87.5 ± 2.2% (EM-NLCs4). The minimum encapsulation is shown by the composition lipid (6%), surfactant (2%) and sonication time (2.5 min), and the maximum is shown by the composition lipid (6%), surfactant (5%) and sonication time (2.5 min). The result exhibited a significant variation in the encapsulation efficiency after varying the NLCs composition. The three-dimensional response surface plot (Figure 3) and the polynomial Equation (5) depicted the effect of independent variables on the EE (Y_2_). The factors lipid (A), surfactant (B) and sonication time (C) showed a positive influence on the encapsulation efficiency. The increase in lipid concentration led to an increase in EE, as depicted in formulations EM-NLCs7 and EM-NLCs8, at a fixed level of surfactant (B) and sonication time (C). The possible reason for this effect is the availability of more space to accommodate the lipophilic drug within the NLCs. However, if the amount of lipid was increased beyond an optimum value (or insufficient amount of surfactant), the EE decreased, as found in EM-NLCs1 and EM-NLCs2 [40]. Similarly, in the presence of sufficient lipid with increased surfactant concentration, the EE also increased (EM-NLCs1 and EM-NLCs 14). This might be due to the higher solubility of the drug in the lipid and the presence of sufficient surfactant. On the other hand, at lower lipid content, the reverse results will cause a reduction in EE (EM-NLCs1 and EM-NLCs3). This effect may be due to the leakage of a drug into the external phase, reducing the EE. The third factor sonication time depicted the enhancement in EE at longer sonication duration (EM-NLCs1 and EM-NLCs5). This might be due to the high energy provided by sonication helping to prevent the drug from leaching from the NLCs system, thus increasing the EE [27]. The second-order polynomial generated for entrapment efficiency was found to be:

Entrapment efficiency (Y_2_) = 82.61 + 4.00A + 1.93B + 0.92C + 3.61AB − 1.14AC − 2.14BC − 1.88A^2^ − 2.11B^2^ − 1.88C^2^ (5)

The independent variables exhibited a positive effect on entrapment efficiency (Y_2_) with the quadratic (R^2^ = 0.9913) model as the best fit model. The lack of fit was found to be insignificant (*p* = 0.6171) with F-value of 2.67. The regression values of the all model for each response are shown in Table 3. The regression coefficient (R^2^) of the quadratic model was found to be 0.9913, indicating the good correlation between response and selected factors. A close agreement in the regression value was observed for the actual and predicted quadratic model. 

### 4.6. Point Prediction

**Figure 4:**
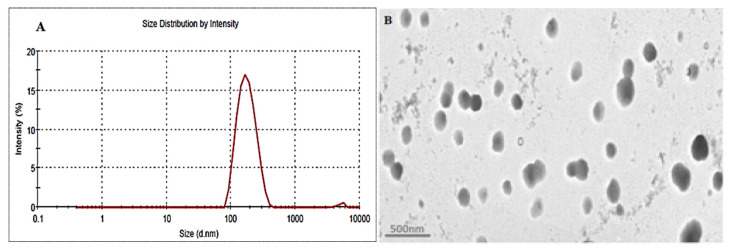
(**A**). Particle size and (**B**). TEM image of EM-NLCs-opt.

The optimized formulation (EM-NLCs-opt) was selected from the point prediction method after slight modification in the independent variables and their effect was observed on PS (Y_1_) and EE (Y_2_), as shown in Table 4. The optimum composition of EM-NLCs-opt formulation is found as lipid (3.5%), surfactant (4%) and sonication time (5.5 min). The actual value of PS and EE of EM-NLCs-opt formulation was found to be 169.6 ± 4.8 nm (Figure 4A), and 81.7 ± 1.4%, respectively. The predicted PS and EE were found to be 167.4 nm and 83.8%, respectively. The close agreement between the practical and predicted value confirms the validation of the model. PDI (0.14) and ZP (−23 mV) values of the optimized formulation were found under the standard limit. The practical value of PDI (<0.5) and ZP (±30 mV) indicates that prepared NLCs have shown homogeneous distribution and high stability. The value lower and greater than the standard gives the unstable composition. The morphology of EM-NLCs-opt was evaluated by TEM and the image showed spherical shape particles with uniform distribution (Figure 4B). There was no aggregation observed, which further supports the PDI evaluation. 

### 4.7. Development of EM-NLCs In Situ Gel

The optimized formulation (EM-NLCs-opt) was converted to in situ gel (EM-NLCs-opt-IG) using different concentrations of carbopol and a fixed concentration of chitosan. The prepared gels were evaluated for different parameters to select the optimum gelling agent concentration (Table 5).

### 4.8. Characterization of EM-NLCs In Situ Gel

#### 4.8.1. Gelling Strength

Gelling strength is an important parameter for the in situ gel system as it directly influences the residence time in the ocular region. It is indicated by gel formation on contact with STF at physiological pH and remains stable for a long time. The gelling capacity of different EM-NLCs-IG formulations is shown in Table 5. The different concentrations of gelling agents (carbopol and chitosan) were used to prepare EM-NLCs-opt-IG1 to EM-NLCs-opt-IG5 and the gelling strength was represented by the negative and positive signs. EM-NLCs-opt-IG1 showed no gelation, EM-NLCs-opt-IG2, EM-NLCs-opt-IG3 indicates slight gelation but have different gelation times. EM-NLCs-opt-IG2 showed gelation time of 3 min but disappeared after a few minutes (<10 min) and EM-NLCs-opt-IG3 showed gelation quickly (5 s) and stable for 2 h. However, EM-NLCs-opt-IG4 exhibited quick gelation (5 s) and were stable for more than 24 h and EM-NLCs-opt-IG5 depicted the gelation time (<5 s), and it forms the thick gel and was found stable for more than 24 h. From the result, it was observed that with the increase in the concentration of carbopol the gelling power increases. Gel formation took place by electrostatic repulsion between an ionic functional group of polymers (pH-sensitive) on increasing the pH when in contact with tear fluid [41,42]. 

#### 4.8.2. Viscosity Measurement

Due to the high shear rate during the blinking (0.03 S^−1^) of the eyelid, most of the administered formulation dose was eliminated from the cul-de-sac [43]. So, the optimum viscosity of the in situ gel system needed was not affected by the shear rate and tear fluid flow. Viscosity also influenced the gelling strength and residence time, in addition to prolonged releases. The result of the viscosity of EM-NLCs-opt-IGs formulations in normal and physiological conditions (STF) is shown in Table 5. The result of the study showed that the viscosity of formulation increases with the increase in carbopol concentration at a fixed chitosan concentration in both conditions (normal and STF). The viscosity of EM-NLCs-opt-IG4 prepared with carbopol (0.4%) and chitosan (0.4%) increased after adding it into STF, due to the crosslinking of the polymer with the ion present in STF [44]. The EM-NLCs-opt-IG4 exhibited the viscosity of 216.32 ± 1.35 cp with STF, and the gel remained stable for more than 24 h, hence it was selected as the optimized formulation. 

#### 4.8.3. In Vitro Drug Release Study

**Figure 5:**
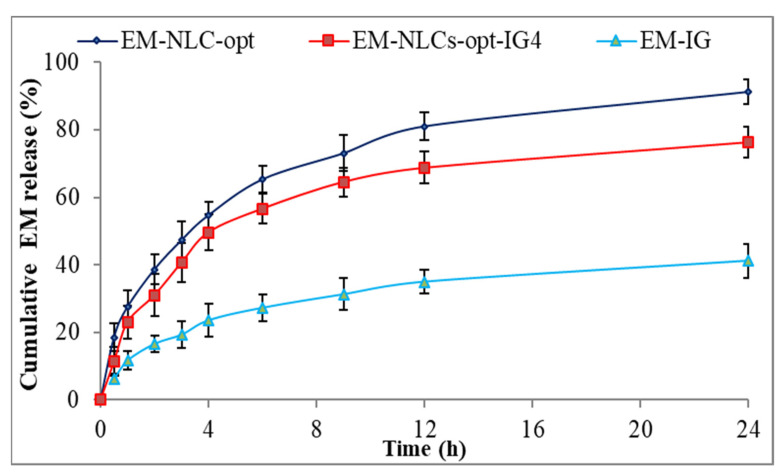
In vitro drug release profile of EM-NLCs-opt, EM-NLCs-opt-IG4, and EM-in situ gel (EM-IG). The study performed in triplicate and data shown as mean ± SD.

The comparative drug release study of EM-NLCs-opt, EM-NLCs-opt-IG4 and EM-IG was performed, and the result is shown graphically in Figure 5. The release order of EM was found as EM-NLCs-opt (65.3 ± 3.9%) > EM-NLCs-opt-IG4 (56.6 ± 4.4%) > EM-in situ gel (27.32 ± 4.1%) in 6 h. However, EM-NLCs-opt, EM-NLCs-opt-IG4, EM-IG exhibited sustained release, i.e., 91.3 ± 3.8%, 76.3 ± 4.5% and 41.2 ± 5.1% in 24 h. The formulation EM-NLCs-opt showed a higher EM release than EM-NLCs-opt-IG4. This may be due to surface deposition of EM on the surface of NLCs, as well as the direct contact of EM-NLCs with release media. However, EM-NLCs-opt-IG4 showed slightly lower EM release than EM-NLCs-opt due to the presence of an extra layer of gelling agent on the NLCs. The drug (EM) needs to cross the gel matrix, so the lesser EM releases. The slower release of EM is ideal for the ophthalmic topical formulation, which helps to achieve better therapeutic activity. EM-NLCs-opt and EM-NLCs-opt-IG4 showed significantly higher release of EM due to the increase in the solubility of EM in the presence of the lipid and surfactant. The drug release from EM-IG was found to be significantly low due to the poor solubility of EM, high viscosity, and formation of insoluble gel matrix hindering the release of EM in STF media [45]. The release data of the EM-NLCs-opt-IG4 were fitted in different kinetic models to determine the best fit model. The regression coefficient (R^2^) of kinetic model, i.e., zero-order (R² = 0.7039), first-order (R² = 0.8435), Higuchi (R² = 0.8887), Korsmeyer—Peppas model (R² = 0.9269) and Hixon—Crowell model (R² = 0.8185). The maximum R^2^ value was found to be for the Korsmeyer—Peppas model and considered as the best fit model. The *n* = 0.48 (0.43 < *n* < 0.85) indicated non-Fickian diffusion (anomalous) mechanism of drug release [46].

#### 4.8.4. Mucoadhesive Study

The mucoadhesive force of EM-NLCs-opt-IG4 was determined by the physical balance method and the value was found to be 1087.38 dyne/cm^2^. The value was found to be significantly higher than the tear film shear force (150 dyne/cm^2^). The high mucoadhesiveness of EM-NLCs-opt-IG4 is due to the combination of gelling polymers (carbopol and chitosan). It indicates that EM-NLCs-opt-IG4 have a high residence time and are not eliminated by tear fluid flow and blinking. 

#### 4.8.5. Ex Vivo Corneal Permeation Study

**Figure 6:**
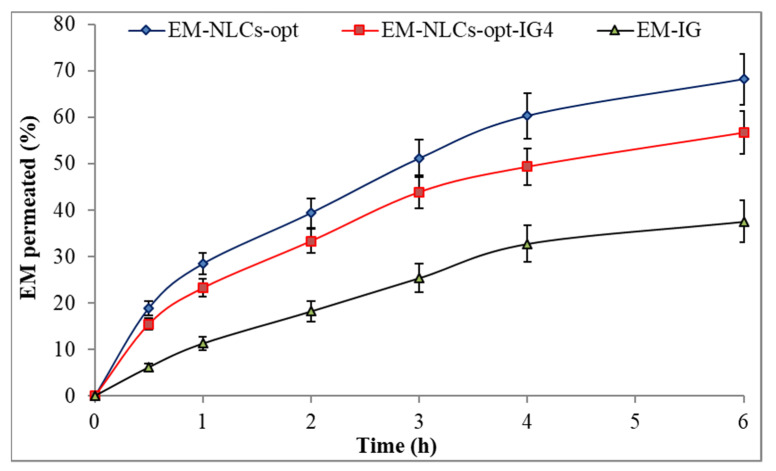
Ex vivo corneal permeation of EM-NLCs-opt, EM-NLCs-opt-IG4 and EM-In situ gel (EM-IG). The study performed in triplicate and data shown as mean ± SD.

Figure 6 shows the ex-vivo permeation result of the EM-NLCs-opt, EM-NLCs-opt-IG4 and EM-IG on goat cornea. EM corneal permeation (%) was found to be 68.2 ± 5.4% from EM-NLCs-opt, 56.7 ± 4.5% from EM-NLCs-opt-IG4 and 37.6 ± 4.5% from EM-IG. The highly significant (*p* < 0.001) permeation was achieved from EM-NLCs-opt and EM-NLCs-opt-IG4 than EM-IG. A significant (*p* < 0.05) difference was observed between EM-NLCs-opt and EM-NLCs-opt-IG4. The permeation data was also used to calculate the flux. The result showed the flux value of 172.15 µg cm^−2^/h from EM-NLCs-opt, 143.24 µg cm^−2^/h from EM-NLCs-opt-IG4 and 94.91 µg cm^−2^/h from EM-IG. EM-NLCs-opt-IG4 exhibited lesser permeation and flux than EM-NLCs-opt due to the formation of a gel network matrix in STF. However, EM-NLCs-opt-IG4 exhibited significant (*p* < 0.05) high permeation and flux than EM-IG. The high permeation and flux of EM-NLCs-opt-IG4 than EM-IG is due to nano-metric size, presence of surfactant (helps to solubilize), presence chitosan (bioadhesive polymer) and carbopol (helps to open the tight junction of lipid membrane). The corneal permeation enhances due to the increase in residence time and prevents the loss of drugs by tear fluid. It can also develop a film over corneal epithelium and release the drug slowly [47]. Nano-size range of particles can be internalized in the carnal cell by a receptor-mediated endocytosis uptake mechanism that can increase the permeation [16].

#### 4.8.6. Histopathological Examination

**Figure 7:**
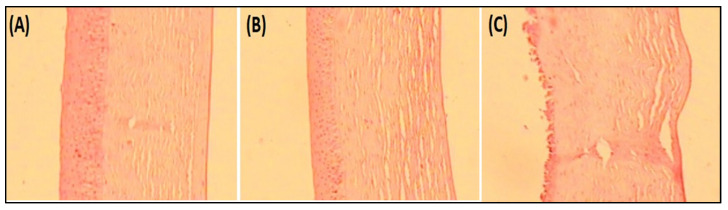
Histopathological image of (**A**). EM-NLCs-opt-IG4, (**B**). Negative control (0.9% NaCl) and (**C**). Positive control as SLS (1% *w*/*v*).

Histopathology study on goat cornea showed the cornea treated with EM-NLCs-opt-IG4 and negative control (0.9% NaCl) had typical corneal structures (Figure 7A,B), and cells retained their normal morphology. It was concluded that, upon treatment, the epidermal layer remains unchanged. The cornea treated with the positive control (1% *w*/*v*; SLS) showed damage to the destruction of corneal epithelial cells (Figure 7C). Finally, based on histopathology study, it was established that the EM-NLCs-opt-IG4 was found safe for ocular delivery.

#### 4.8.7. Corneal Hydration 

This study determined the damage in goat cornea after the application of the formulation. EM-NLCs-opt-IG4 treated cornea showed a hydration value of 79.34% after the study. The value was found to be within the standard limit of 75–80% [48]. So, from the study, we can say that the developed formulation was found to be safe for ocular administration.

#### 4.8.8. HET CAM Irritation Study

**Figure 8:**
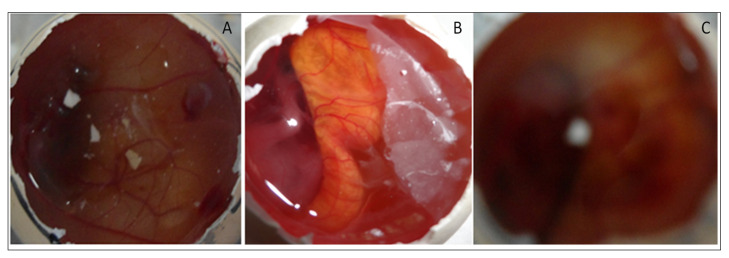
HET-CAM image of (**A**). EM-NLCs-opt-IG4, (**B**). Negative control (0.9% NaCl) and (**C**). Positive control (0.1 N NaOH).

HET-CAM (irritation) study was performed for EM-NLCs-opt-IG4, normal saline (0.9% NaCl, negative control) and positive control 0.1N sodium hydroxide) using a fertilized hen egg’s (Figure 8). The developed CAM after incubation is similar to the artery and vein of an eye. EM-NLCs-opt-IG4 and normal saline-treated CAM showed no irritation. There was no haemorrhage and bleeding from the artery and vein of CAM (no breaking) observed in 5 min. and the score was found to be closer to zero. In addition, the positive control (0.1N NaOH) treated CAM depicted a score of 6 and indicated haemorrhage of artery and vein of CAM in 5 min. So, the positive control treated CAM is considered a severe irritant. The study revealed that the developed EM-NLCs-opt-IG4 formulation was found to be non-irritant and safe for ophthalmic administration. 

#### 4.8.9. Sterility and Isotonicity Study 

**Figure 9:**
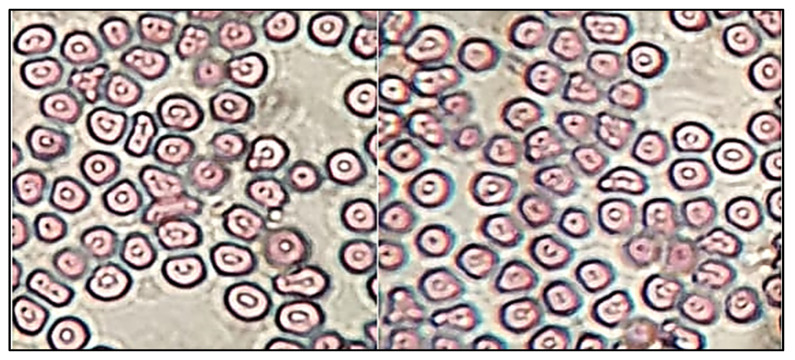
Isotonicity image of EM-NLCs-opt-IG4 and normal saline treated blood cells.

The sterility test was performed to evaluate the microbial growth in EM-NLCs-opt-IG4. The sterility study result showed no microbial growth in fluid thioglycolate and soybean casing digest media after placing the formulation for 14 days. Figure 9 depicted the isotonicity test of EM-NLCs-opt-IG4 and the result compared with the normal saline (0.9% NaCl). No damage in red blood cells was observed (shrinking and swelling) after treatment with EM-NLCs-opt-IG4. The result was found to be the same for normal saline-treated cells. From the study, it can be revealed that EM-NLCs-opt-IG4 formulation was found to be isotonic. 

#### 4.8.10. Antimicrobial Activity

**Figure 10:**
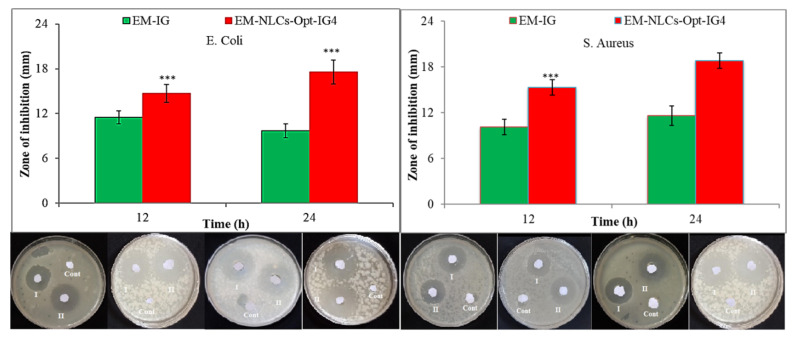
Comparative antimicrobial results of EM-in situ gel (EM-IG) and EM-NLCs-opt-IG4 against *S. aureus* and *E. coli* at 12 h and 24 h study. The study performed in triplicate and data shown as mean ± SD. *** *p* < 0.001 considered as highly significant than EM in situ gel.

Antibacterial evaluation study EM-NLCs-opt-IG4 and normal EM-IG were assessed on Gram-positive bacteria (*S. aureus*) and Gram-negative (*E. coli*) bacterial the using cup plate method and data was depicted in Figure 10. EM-NLCs-opt-IG4 exhibited ZOI of 16 ± 1 mm (12 h) and 19 ± 1 mm (24 h) against *S. aureus*. EM-IG showed ZOI of 10 ± 1 mm (12 h) and 13 ± 2 mm (24 h) against *S. aureus*. However, EM-NLCs-opt-IG4 showed ZOI against *E. coli* is of 17 ± 1 mm and 21 ± 2 mm in 12 h and 24 h, respectively. Furthermore, EM-IG showed ZOI against *E. coli* is 11 ± 1 mm and 14 ± 2 mm in 12 h and 24 h, respectively. The results revealed that EM-NLCs-opt-IG4 exhibited significantly (*p* < 0.001) higher activity than EM-IG due to more solubility of EM in the NLCs gel system due to the presence of the lipid and surfactant. This may enhance the permeation to cell wall of bacteria and inhibited protein synthesis by arresting ribosome. It was also observed that EM is more effective in Gram-negative bacteria than Gram-positive bacteria. 

## 5. Conclusions

Erythromycin loaded NLCs were successfully prepared by the emulsification ultrasonication method and further optimized by central composite design using lipid (stearic acid+ oleic acid), surfactant (Pluronic F127) and sonication time. EM-NLCs-opt showed nano-metric size with high entrapment efficiency. EM-NLCs-opt formulation was successfully incorporated in situ gel using pH-sensitive carbopol 940 and chitosan. EM-NLCs-opt-IG4 showed frequent gelation, stable for more than 24 h and also depicted significantly enhanced bioadhesion. EM-NLCs-opt-IG4 showed a sustained release profile with high ex vivo goat permeation than EM in situ gel. It also exhibited good tolerability and irritation established by the hydration study, histopathology and HET-CAM test. EM-NLCs-opt-IG4 showed better antimicrobial activity due to the nano-size of the NLCs and high release of EM. It was concluded that NLCs incorporated in situ gel are a better alternative carrier for the improvement of precorneal residence time and therapeutic efficacy.

## Figures and Tables

**Table 1:** Independent factors and their levels with the dependent factors used in the central composite design.

Independent Factors	Units	Levels			Star Points	
		Low (−1)	Medium (0)	High (+1)	−α (−1.68)	+α (+1.68)
Lipid (A)	(%)	2	4	6	0.64	7.36
Surfactant (B)	(%)	2	3.5	5	0.98	6.02
Sonication time (C)	(min)	2.5	5	7.5	0.8	9.2
**Dependent factors**	Goals					
Particle size	(nm)	Minimum				
Entrapment efficiency	(%)	Maximum				

**Table 2:** Composition of various batches and their predicted values obtained from central composite design along with their experimental values of EM-NLCs.

Formulation	Lipid (%)	Surfactant (%)	Sonication Time (min)	Particle Size (nm)	Entrapment Efficiency (%)
	A	B	C	Y_1_	Y_2_
EM-NLCs1	2	2	2.5	239.2 ± 5.2	73.5 ± 1.2
EM-NLCs2	6	2	2.5	350.6 ± 4.2	69.4 ± 1.3
EM-NLCs3	2	5	2.5	219.5 ± 6.2	71.5 ± 1.6
EM-NLCs4	6	5	2.5	261.5 ± 4.9	87.5 ± 2.2
EM-NLCs5	2	2	7.5	199.7 ± 3.7	78.5 ± 1.5
EM-NLCs6	6	2	7.5	319.4 ± 7.6	76.5 ± 1.9
EM-NLCs7	2	5	7.5	122.6 ± 6.1	70.5 ± 1.4
EM-NLCs8	6	5	7.5	201.3 ± 7.2	84.5 ± 1.6
EM-NLCs9	0.64	3.5	5	140.6 ± 3.4	70.5 ± 1.7
EM-NLCs10	7.36	3.5	5	285.3 ± 6.2	84.5 ± 1.9
EM-NLCs11	4	0.98	5	346.9 ± 7.9	73.5 ± 1.4
EM-NLCs12	4	6	5	177.3 ± 5.2	80.2 ± 1.6
EM-NLCs13	4	3.5	0.8	287.4 ± 6.3	76.5 ± 1.8
EM-NLCs14	4	3.5	9.2	177.5 ± 5.3	78.5 ± 1.4
* EM-NLCs15	4	3.5	5	195.5 ± 3.6	82.6 ± 1.2
* EM-NLC16	4	3.5	5	195.5 ± 3.6	82.6 ± 1.2
* EM-NLC17	4	3.5	5	195.5 ± 3.6	82.6 ± 1.2
* EM-NLC18	4	3.5	5	195.5 ± 3.6	82.6 ± 1.2
* EM-NLC19	4	3.5	5	195.5 ± 3.6	82.6 ± 1.2
* EM-NLC20	4	3.5	5	195.5 ± 3.6	82.6 ± 1.2

* Centre point having same composition.

**Table 3:** Regression values of the selected responses during optimization.

Model	Y_1_ (Particle Size, nm)				
	SD	R²	Adjusted R²	Predicted R²	*p*-Value
Linear	31.8	7909	0.7517	0.6648	0.0073
2FI	29.8	8496	0.7802	0.5737	0.0198
Quadratic	11.6	0.9827	0.9671	0.8997	0.0001
	**Y_2_ (Entrapment efficiency, %)**				
Linear	4.29	0.4887	0.3929	0.1700	<0.0001
2FI	2.32	0.7512	0.6364	0.2857	<0.0001
Quadratic	0.71	0.9913	0.9836	0.9252	<0.0011

**Table 4:** Point prediction optimization by central composite design.

Formulations	Lipid: Surfactant: Sonication Time	Actual Value		Predicted Value	
		Y_1_ (nm)	Y_2_ (%)	Y_1_ (nm)	Y_2_ (%)
EM-NLC-opt	3.5:4:5.5	169.6 ± 4.8	81.7 ± 1.4	167.4	83.8

**Table 5:** Physicochemical parameter evaluation of EM-NLCs in situ gel system. The study performed in triplicate and data shown as mean ± SD.

Code	Carbopol (%)	Chitosan (%)	pH	Optical Transmittance (%)	Drug Content (%)	Gelling Strength	Viscosity (cP)	
							Solution	With STF
EM-NLCs-opt2-IG1	0.1	0.4	6.54 ± 0.4	94.8 ± 1.1	98.7 ± 3.1	−	6.2 ± 1.1	10.5 ± 2.1
EM-NLCs-opt2-IG2	0.2	0.4	6.43 ± 0.6	95.8 ± 0.8	98.8 ± 2.1	+	38.5 ± 3.7	141.3 ± 2.4
EM-NLCs-opt2-IG3	0.3	0.4	6.24 ± 0.2	95.7 ± 1.1	98.2 ± 2.7	++	50.4 ± 1.7	176.4 ± 2.5
EM-NLCs-opt2-IG4	0.4	0.4	6.16 ± 0.2	98.5 ± 0.4	99.6 ± 3.7	+++	87.4 ± 2.3	216.3 ± 1.3
EM-NLCs-opt2-IG5	0.5	0.4	6.02 ± 0.7	96.6 ± 0.9	99.6 ± 1.4	++++	98.5 ± 2.1	254.8 ± 1.1

(−) no gel formation, (+) gel formation in 3 min and disappeared <10 min, (++) gel formation in <5 s and disappeared after 2 h, (+++) gel formation in <5 s and stable for >24 h, (++++) gel formation in <5 s, hard gel and stable for >24 h.

## Data Availability

Not applicable.

## References

[1] Gholizadeh S., Wang Z., Chen X., Dana R., Annabi N. (2021). Advanced nanodelivery platforms for topical ophthalmic drug delivery. Drug Discov. Today.

[2] Baig M.S., Ahad A., Aslam M., Imam S.S., Aqil M., Ali A. (2016). Application of Box-Behnken design for preparation of levofloxacin-loaded stearic acid solid lipid nanoparticles for ocular delivery: Optimization, in vitro release, ocular tolerance, and antibacterial activity. Int. J. Biol. Macromol..

[3] Agrawal A., Das M., Jain S. (2012). In situ gel systems as ’smart’ carriers for sustained ocular drug delivery. Expert Opin. Drug Deliv..

[4] Maulvi F.A., Shetty K.H., Desai D.T., Shah D.O., Willcox M.D.P. (2021). Recent advances in ophthalmic preparations: Ocular barriers, dosage forms and routes of administration. Int. J. Pharm..

[5] Lakhani P., Patil A., Wu K.W., Sweeney C., Tripathi S., Avula B., Taskar P., Khan S., Majumdar S. (2019). Optimization, stabilization, and characterization of amphotericin B loaded nanostructured lipid carriers for ocular drug delivery. Int. J. Pharm..

[6] Ferreira K.S.A., Santos B.M.A.D., Lucena N.P., Ferraz M.S., Carvalho R.S.F., Duarte Júnior A.P., Magalhães N.S.S., Lira R.P.C. (2018). Ocular delivery of moxifloxacin-loaded liposomes. Arq. Bras. Oftalmol..

[7] Ameeduzzafar, Alruwaili N.K., Imam S.S., Alotaibi N.H., Alhakamy N.A., Alharbi K.S., Alshehri S., Afzal M., Alenezi S.K., Bukhari S.N.A. (2020). Formulation of Chitosan Polymeric Vesicles of Ciprofloxacin for Ocular Delivery: Box-Behnken Optimization, In Vitro Characterization, HET-CAM Irritation, and Antimicrobial Assessment. AAPS PharmSciTech.

[8] Taghe S., Mirzaeei S., Alany R.G., Nokhodchi A. (2020). Polymeric Inserts Containing Eudragit^®^ L100 Nanoparticle for Improved Ocular Delivery of Azithromycin. Biomedicines.

[9] Shelley H., Rodriguez-Galarza R.M., Duran S.H., Abarca E.M., Babu R.J. (2018). In Situ Gel Formulation for Enhanced Ocular Delivery of Nepafenac. J. Pharm. Sci..

[10] Janagam D., Wu L., Lowe T. (2017). Nanoparticles for drug delivery to the anterior segment of the eye. Adv. Drug Deliv. Rev..

[11] Zhang W., Li X., Ye T., Chen F., Sun X., Kong J., Yang X., Pan W., Li S. (2013). Design, characterization, and in vitro cellular inhibition and uptake of optimized genistein-loaded NLC for the prevention of posterior capsular opacification using response surface methodology. Int. J. Pharm..

[12] Liu D., Li J., Pan H., He F., Liu Z., Wu Q., Bai C., Yu S., Yang X. (2016). Potential advantages of a novel chitosan-N-acetylcysteine surface modified nanostructured lipid carrier on the performance of ophthalmic delivery of curcumin. Sci. Rep..

[13] El-Salamouni N.S., Farid R.M., El-Kamel A.H., El-Gamal S.S. (2018). Nanostructured lipid carriers for intraocular brimonidine localisation: Development, in-vitro and in-vivo evaluation. J. Microencapsul..

[14] Abd-Elhakeem E., El-Nabarawi M., Shamma R. (2021). Lipid-based nano-formulation platform for eplerenone oral delivery as a potential treatment of chronic central serous chorioretinopathy: In-vitro optimization and ex-vivo assessment. Drug Deliv..

[15] Tavakoli N., Taymouri S., Saeidi A., Akbari V. (2019). Thermosensitive hydrogel containing sertaconazole loaded nanostructured lipid carriers for potential treatment of fungal keratitis. Pharm. Dev. Technol..

[16] Youssef A., Dudhipala N., Majumdar S. (2020). Ciprofloxacin Loaded Nanostructured Lipid Carriers Incorporated into In-Situ Gels to Improve Management of Bacterial Endophthalmitis. Pharmaceutics.

[17] Gade S., Patel K.K., Gupta C., Anjum M.M., Deepika D., Agrawal A.K., Singh S. (2019). An Ex Vivo Evaluation of Moxifloxacin Nanostructured Lipid Carrier Enriched In Situ Gel for Transcorneal Permeation on Goat Cornea. J. Pharm. Sci..

[18] Freitas P.R., de Araújo A.C.J., Barbosa C.R., Muniz D.F., Tintino S.R., Ribeiro-Filho J., Siqueira Júnior J.P., Filho J.M.B., de Sousa G.R., Coutinho H.D.M. (2021). Inhibition of Efflux Pumps by Monoterpene (α-pinene) and Impact on *Staphylococcus aureus* Resistance to Tetracycline and Erythromycin. Curr. Drug Metab..

[19] Olajuyigbe O.O., Animashaun T. (2012). Synergistic activities of amoxicillin and erythromycin against bacteria of medical importance. Pharmacologia.

[20] Gupta S., Vyas S.P. (2010). Carbopol/chitosan based pH triggered in situ gelling system for ocular delivery of timolol maleate. Sci. Pharm..

[21] Wu Y., Liu Y., Li X., Kebebe D., Zhang B., Ren J., Lu J., Li J., Du S., Liu Z. (2019). Research progress of in-situ gelling ophthalmic drug delivery system. Asian J. Pharm. Sci..

[22] Sheshala R., Kok Y.Y., Ng J.M., Thakur R.R., Dua K. (2015). In situ gelling ophthalmic drug delivery system: An overview and its applications. Recent Pat. Drug Deliv..

[23] Kong M., Chen X.-G., Xing K., Park H.-J. (2010). Antimicrobial properties of chitosan and mode of action: A state of the art review. Int. J. Food Microb..

[24] Modi D., Mohammad Warsi M.H., Garg V., Bhatia M., Kesharwani P., Jain G.K. (2021). Formulation development, optimization, and in vitro assessment of thermoresponsive ophthalmic pluronic F127-chitosan *in situ* tacrolimus gel. J. Biomater. Sci. Polym. Ed..

[25] Cirri M., Maestrini L., Maestrelli F., Mennini N., Mura P., Ghelardini C., Di Cesare Mannelli L. (2018). Design, characterization and in vivo evaluation of nanostructured lipid carriers (NLC) as a new drug delivery system for hydrochlorothiazide oral administration in pediatric therapy. Drug Deliv..

[26] Hao J., Wang F., Wang X., Zhang D., Bi Y., Gao Y., Zhao X., Zhang Q. (2012). Development and optimization of baicalin-loaded solid lipid nanoparticles prepared by coacervation method using central composite design. Eur. J. Pharm. Sci..

[27] Kollipara S., Bende G., Movva S., Saha R. (2010). Application of rotatable central composite design in the preparation and optimization of poly(lactic-co-glycolic acid) nanoparticles for controlled delivery of paclitaxel. Drug Dev. Ind. Pharm..

[28] Ye Q., Li J., Li T., Ruan J., Wang H., Wang F., Zhang X. (2021). Development and evaluation of puerarin-loaded controlled release nanostructured lipid carries by central composite design. Drug Dev. Ind. Pharm..

[29] Velmurugan R., Selvamuthukumar S. (2016). Development and optimization of ifosfamide nanostructured lipid carriers for oral delivery using response surface methodology. Appl. Nanosci..

[30] Makoni P.A., Khamanga S.M., Walker R.B. (2021). Muco-adhesive clarithromycin-loaded nanostructured lipid carriers for ocular delivery: Formulation, characterization, cytotoxicity and stability. J. Drug Deliv. Sci. Technol..

[31] Tavakoli M., Mahboobian M.M., Nouri F., Mohammadi M. (2021). Studying the ophthalmic toxicity potential of developed ketoconazole loaded nanoemulsion *in situ gel* formulation for ophthalmic administration. Toxicol. Mech. Methods.

[32] Katiyar S., Pandit J., Mondal R.S., Mishra A.K., Chuttani K., Aqil M., Ali A., Sultana Y. (2014). In situ gelling dorzolamide loaded chitosan nanoparticles for the treatment of glaucoma. Carbohydr. Polym..

[33] Upadhayay P., Kumar M., Pathak K. (2016). Norfloxacin Loaded pH Triggered Nanoparticulate in-situ Gel for Extraocular Bacterial Infections: Optimization, Ocular Irritancy and Corneal Toxicity. Iran. J. Pharm. Res..

[34] Wardrop J., Ficker D., Franklin S., Gorski R.J. (2000). Determination of erythromycin and related substances in enteric-coated tablet formulations by reversed-phase liquid chromatography. J. Pharm. Sci..

[35] Nagarwal R.C., Kumar R., Pandit J.K. (2012). Chitosan coated sodium alginate-chitosan nanoparticles loaded with 5-FU for ocular delivery: In vitro characterization and in vivo study in rabbit eye. Eur. J. Pharm. Sci..

[36] Bagley D.M., Waters D., Kong B.M. (1994). Development of a 10-day chorioallantoic membrane vascular assay as an alternative to the Draize rabbit eye irritation test. Food Chem. Toxicol..

[37] (2010). ICCVAM-Recommended Test Method Protocol: Hen’s Egg Test—Chorioallantoic Membrane (HET-CAM) Test Method. ICCVAM Test Method Eval. Rep..

[38] Hiremath S.S.P., Dasankoppa F.S., Nadaf A., Jamakandi V.G., Mulla J.S., Sholapur H.N., Aezaz A. (2008). Formulation and Evaluation of a Novel *In Situ* Gum Based Ophthalmic Drug Delivery System of Linezolid. Sci. Pharm..

[39] Yang G., Wu F., Chen M., Jin J., Wang R., Yuan Y. (2019). Formulation design, characterization, and in vitro and in vivo evaluation of nanostructured lipid carriers containing a bile salt for oral delivery of gypenosides. Int. J. Nanomed..

[40] Behbahani E.S., Ghaedi M., Abbaspour M., Rostamizadeh K. (2017). Optimization and characterization of ultrasound assisted preparation of curcumin-loaded solid lipid nanoparticles: Application of central composite design, thermal analysis and X-ray diffraction techniques. Ultrason. Sonochem..

[41] Tinu T.S., Thomas L., Kumar A. (2013). Polymers used in ophthalmic in situ gelling system. Int. J. Pharm. Sci. Rev. Res..

[42] Hamman J.H. (2010). Chitosan based polyelectrolyte complexes as potential carrier materials in drug delivery systems. Mar. Drugs..

[43] Abraham S., Furtado S., Bharath S., Basavaraj B.V., Deveswaran R., Madhavan V. (2009). Sustained ophthalmic delivery of ofloxacin from an ion-activated in situ gelling system. Pak. J. Pharm. Sci..

[44] Shukr M.H., Ismail S., El-Hossary G.G., El-Shazly A.H. (2021). Design and evaluation of mucoadhesive in situ liposomal gel for sustained ocular delivery of travoprost using two steps factorial design. J. Drug Deliv. Sci. Technol..

[45] Fahmy U.A., Ahmed O.A.A., Badr-Eldin S.M., Aldawsari H.M., Okbazghi S.Z., Awan Z.A., Bakhrebah M.A., Alomary M.N., Abdulaal W.H., Medina C. (2020). Optimized Nanostructured Lipid Carriers Integrated into In Situ Nasal Gel for Enhancing Brain Delivery of Flibanserin. Int. J. Nanomed..

[46] Yu S., Li Q., Li Y., Wang H., Liu D., Yang X., Pan W. (2017). A novel hydrogel with dual temperature and pH responsiveness based on a nanostructured lipid carrier as an ophthalmic delivery system: Enhanced trans-corneal permeability and bioavailability of nepafenac. New J. Chem..

[47] Ritger P.L., Peppas N.A. (1987). A simple equation for description of solute release I. Fickian and non-fickian release from non-swellable devices in the form of slabs, spheres, cylinders or discs. J. Control. Release.

[48] Fouda N.H., Abdelrehim R.T., Hegazy D.A., Habib B.A. (2018). Sustained ocular delivery of Dorzolamide-HCl via proniosomal gel formulation: In-vitro characterization, statistical optimization, and in-vivo pharmacodynamic evaluation in rabbits. Drug Deliv..

